# Aggression in Tephritidae Flies: Where, When, Why? Future Directions for Research in Integrated Pest Management

**DOI:** 10.3390/insects6010038

**Published:** 2014-12-30

**Authors:** Giovanni Benelli

**Affiliations:** Insect Behavior Group, Department of Agriculture, Food and Environment, University of Pisa, via del Borghetto 80, 56124 Pisa, Italy; E-Mail: g.benelli@sssup.it or benelli.giovanni@gmail.com; Tel.: +39-502216141; Fax: +39-502216087

**Keywords:** aggressive behavior, communication channels, contest, fighting experience, lateralization, learning, mass-rearing optimization, olfactory cues, true fruit flies

## Abstract

True fruit flies (Diptera: Tephritidae) include over 4000 species, many of which constitute enormous threats to fruit and vegetable production worldwide. A number of Tephritidae are lekking species, forming aggregations in which males fight to defend a small territory where they court females and mate. Male-male contests also occur in non-lekking species, characterized by resource defense polygyny. Tephritidae females display agonistic behavior to maintain single oviposition sites and reduce larval competition for food. Here, how, where, when and why aggressive interactions occur in Tephritidae flies is reviewed. A number of neglected issues deserving further research are highlighted, with a special focus on diel periodicity of aggression, cues evoking aggressive behavior, the role of previous experience on fighting success and the evolution of behavioral lateralization of aggressive displays. In the final section, future directions to exploit this knowledge in Integrated Pest Management, with particular emphasis on enhancement of Sterile Insect Technique and interspecific competitive displacement in the field are suggested.

## 1. Introduction

Tephritidae (Diptera), also known as “true fruit flies”, have over 4000 species, many of which constitute enormous threats to fruit and vegetable production throughout the world, causing both quantitative and qualitative losses [1]. Many species of Tephritidae are highly polyphagous and attack a wide array of economically important fruit and vegetable crops including mango, peach, apple, pear, citrus, guava, avocado, tomato, pepper, and cucurbits [2,3], whereas others are oligophagous [4]. Adult female fruit flies can cause direct damage by laying eggs under the skin of fruits and vegetables, then the eggs hatch into larvae that feed on the decaying flesh of the crop. Infested fruits and vegetables quickly become inedible or drop to the ground [5]. Furthermore, due to their susceptibility to invasive Tephritidae species, many fruit-producing countries have imposed quarantine restrictions on the import of products from countries infested with particular fruit fly species, and/or require that fruits and vegetables undergo quarantine treatment before their importation is allowed. Thus, suppression or eradication of true fruit flies has often been the goal of control programs [6,7]. Chemical control (*i.e.*, cover and bait sprays) based on conventional (e.g., organophosphates and pyrethroids) and plant-borne insecticides [4,8,9] as well as biotechnical tools (e.g., Sterile Insect Technique and Male Annihilation Technique) [6,10,11,12], rather than biological control [13,14,15,16], have been the main weapons used in most control programs.

A number of Tephritidae are lekking species, forming aggregations in which males fight to defend a small territory where they court females and mate. Male-male contests also occur in non-lekking species characterized by resource defense polygyny. Tephritidae females display agonistic behavior to maintain single oviposition sites and reduce larval competition for food [11,12]. Here I review how, where, when and why aggressive interactions occur in Tephritidae flies. Neglected issues deserving further research are highlighted, with a special focus on diel periodicity of aggression, cues evoking aggressive behavior, the role of previous experience on fighting performances and the evolution of behavioral lateralization of aggression. In the final section of the manuscript, I propose future directions and potential implication for research in Integrated Pest Management, with particular emphasis on enhancement of Sterile Insect Technique and dynamics of interspecific competitive displacement in the field.

## 2. Male-Male Fighting Behavior

### 2.1. Why Males Fight

Among Tephritidae flies, two different polygynous mating systems have been reported, the male dominance polygyny and the resource defense polygyny. Male-male contests occur in both. Male dominance polygyny is typical of tropical and subtropical polyphagous tephritids, such as a number of *Anastrepha*,* Bactrocera* and *Ceratitis* species where males establish leks for mating purposes [17,18,19,20,21]. Following the definition of Aluja and Birke [22], the “lek” is an aggregation of at least three pheromone-calling males in a non-resource based area, usually from adjacent leaves of a host or non-host plant species. Lekking males fight for territories, and then initiate sexual behavior by releasing long-range pheromones that attract females to behavioral exhibition sites [23,24,25,26,27,28]. Females discriminate among lek participants, probably based on the established male dominance hierarchy [29], and copulate with males performing the best courtship behavior sequence, which includes wing movements combined with olfactory and tactile cues [11,30,31,32]. In the majority of cases, stimuli guiding lekking behavior in Tephritidae are not well identified. However, it has been proposed that both environmental factors (e.g., light intensity and/or photoperiod) and male signaling behavior (e.g., wing movements coupled with the release of long-distance pheromones) could be involved at the same time [23,24,26,33,34,35,36].

Why do tephritid females join lekking males? Females mating in leks can achieve direct benefits (e.g., guaranteed fertility, less risk of copulation interruption, avoidance of predators, and reduced probability of acquiring diseases) [37,38,39], and also indirect Fisherian benefits associated with the quality of the genetic resources to be donated to their offspring (the “good genes” hypothesis, or the “Fisher” model) [37,40,41,42]. On the other hand, there are some costs to females participating in leks that should be taken into account. Female flies have a large number of potential males to evaluate as possible sexual partners [43], they can lose potential mates to competitors, and have extensive investment of time and metabolic energy in mating activities [40]. Thus, it appears conceivable that the evolution of female mate preferences is shaped by a balance between the costs and benefits [44].

Resource defense polygyny is typical of many temperate, monophagous or oligophagous Tephritidae, with special reference to the genus *Rhagoletis*. In these flies, evidences of sex pheromones are hard to find, mating occurs on and/or near the host plant, and involves male defense of resources needed by females (*i.e.*, oviposition sites) through male-male aggressions [27,28,45]. Good examples include the apple maggot, *Rhagoletis pomonella* (Walsh) [46], the Western cherry fruit fly, *Rhagoletis indifferens* Curran [47,48], the walnut husk fly, *Rhagoletis completa* Cresson [49], *Rhagoletis suavis* (Loew) [50], *Rhagoletis rubicola* Doane and *Rhagoletis mendax* Curran. In these species males individually seek out females on the fruit surface [48], often while they are engaged in oviposition activities [51,52].

However, within the two polygynous mating systems occurring in Tephritidae, there are some noteworthy exceptions [27,28]. For instance, lekking can occur also in some monophagous species [53]. It has been hypothesized that these are the cases in which the host is sufficiently abundant so that male defense is unlikely to be profitable [54]. On the other hand, the mating behavior of some polyphagous species recalls resource defense polygyny typical of mono- and oligophagous species. For example, the papaya fruit fly, *Toxotrypana curvicauda* Gerstaecker, a tropical species with a narrow host range, produces sex pheromones but apparently does not lek. This species relies on a dual mating system in which males mount ovipositing females [55]. Similarly, another tropical species, *Anastrepha robusta* Greene, produces sex pheromones but, rather than joining in leks, engages male flying loops and repeated landings on the host plant while pheromone calling [56].

In few species, the scenario seems even more complicated, and male dominance polygyny alternates with resource defense at different times of the day or year [28]. For example, males of *Anastrepha suspensa* (Loew) court on fruit in the morning, then signal from the underside of leaves in the late afternoon emitting pheromones and producing wing vibrations. Noticeably, 85% of matings occur on leaves [57]. In *Ceratitis capitata* Wiedemann, males signal from leaves from mid-morning to early afternoon, while they court females on fruits earlier and later. Also in this species, successful matings occurring on leaves are more than twice the ones on fruits [58]. However, calling may lead to higher predation rates [59], and this can provide an explanation for the occurrence of alternative mating strategies in the above-mentioned species [28]. In other species typical of temperate climates, such as the monophagous fly *R. pomonella*, male mating strategy changes seasonally, with males mating on apple tree foliage early in the season and on fruit later in the season, while mating attempts on the two locations achieve comparable success [60].

### 2.2. How Males Fight

In a number of Tephritidae species, male-male aggressive interactions are characterized by reciprocal wing waving (*i.e.*, the attacker faces the opponent and brings both wings forward perpendicular to the longitudinal axis of its body, while the ventral surface of the wings are turned to face the anterior), followed by chasing plus head butting (*i.e.*, lunging at the opponent ending with head butting) and/or boxing with forelegs (*i.e.*, the attacker raises a foreleg, hitting the opponent on the head or thorax) [11,12]. These aggressive displays have been observed both in species where male-male contests occur in non-resource based leks (male dominance polygyny), as well as in species where males fight each other to gain resources crucial to females (resource defense polygyny). Most common examples include *A. ludens* [18,61], *Bactrocera invadens* Drew, Tsuruta & White [62], *Bactrocera oleae* (Rossi) [21], *Ceratitis cosyra* (Walker) [62], *Trupanea jonesi* (Curran) [53], *T. curvicauda* [55], *Aciurina trixa* Curran, *Valentibulla dodsoni* Foote [63], *Procecidochares* sp. [64], *R*. *indifferens* [47,48], *R. pomonella* [46] and *R*. *completa* [49]. Furthermore, some Tephritidae species rely on additional aggressive displays [65]. For instance, fighting males of *C. capitata* also perform labellar contacts and head pushing (*i.e.*, one or both flies extend mouthparts and try to push away the opponent), wing strike acts (*i.e.*, the attacker brings forward a wing and strikes the opponent) and dives (*i.e.*, the attacker flies onto the opponent, hitting it on its dorsum, and retreats quickly) in escalating phases of aggressive encounters [66]. Wing strike is a common aggressive display also in *T. curvicauda* male contests [55]. By contrast, in other tephritids, the sequence of events during male-male combat is less elaborate. In *Bactrocera dorsalis* (Hendel) the male simply runs at the other male and drives it off the leaf, whereas pouncing followed by head butting is rare [67]. In *Paracantha gentilis* Hering, males display agonistic behaviors composed of wing waving, chasing and head butting, although boxing has never been observed [68].

Male-male combat has also been reported for *Bactrocera cucurbitae* Coquillett [17,69], *Trupanea bisetosa* (Coquilett) [70], *Euaresta stigmatica* Coquillett, *Euarestoides acutangulus* (Thomson) and *Tephritis stigmatica* (Coquillett) [53,71,72], but detailed information is not available on the behavioral sequence of these interactions. Noticeably, only in a few species (e.g., *Anastrepha fraterculus* (Wiedemann) and *Ceratitis rosa* (Karsch), male aggressions are not frequent and/or outcomes are not crucial for mating success [35,36,73]. Some lekking studies on tephritid flies (e.g., *A. ludens*,* Procecidochares* sp.) also highlighted the importance of male body size in territory defense and acoustic signaling [61,64,74,75].

Recently, lateralization (*i.e.*, different functional and/or structural specializations of the left and right sides of the brain) of aggressive displays has been found in tephritid flies [66]. *C. capitata* shows left-biased population-level lateralization of aggressive displays (*i.e.*, wing strike and boxing), with no consistent differences among sexes. Aggressive behaviors performed with left body parts lead to greater fighting success over those performed with right body parts. Aggressions that lead to success are faster than unsuccessful ones. However, left wing/legs are not faster than right ones while performing aggressive acts [66]. Lateralization of aggression has been examined in a number of vertebrate species, ranging from cichlids to deer and horses [76,77,78,79], while little is known on invertebrates [80,81,82]. Current theory supports the idea predicted by a theoretical model on the evolution of asymmetries, namely that lateralization at the population-level is more likely to evolve in social species, while lateralization at an individual-level is more likely to evolve in solitary species [77,83,84,85]. Concerning invertebrates, Frasnelli* et al.* [82] reviewed a number of cases in which solitary species (e.g., spiders, water bugs, drosophilid flies, crabs, snails, cuttlefish and squids) show population-level lateralization, and proposed that population-level behavioral asymmetries found in these species are connected with mating and other social interactions (e.g., fighting and escape responses) [82]. This may also be the case for tephritids, where lateralization of aggression at population-level may be connected with prolonged social interactions occurring among males leading to mating, and among females in same-sex contests for preferred oviposition sites [66]. Lateralization of aggressive behavior may be widespread in more than one tephritid species and further research on this point is required for a better understanding of their evolutionary ecology.

### 2.3. Where Males Fight

In tephritids with male dominance polygyny, the position of lek sites varies among different fruit fly species. Leks can occur on various parts of host plants, or even away from host plants [75]. Olive fruit fly males prefer the windward side of olive trees, and perform most male-male aggression behaviors, as well as mating interactions, on olive leaves [21]. *C. capitata* leks occur on various parts of host trees, including fruits, leaves and trunks [19,67]. In contrast, melon fly males gather mainly around non-host trees or weeds, to which the females are attracted; males fight each other on the lower surface of tree leaves or weeds, then stridulates with their wings and releases pheromones [17,69]. Other species, such as the gall-forming tephritid flies *A. trixa* and *V. dodsoni*, do not maintain individual territories and weakly rely on lek dynamics to mate with females [63]. Males of the papaya fruit fly, *T. curvicauda*, prefer papaya fruits as non-lek mating sites [55]. Similarly, in tephritids with resource defense polygyny, such as* Rhagoletis* spp., it has been reported that males usually converge and fight in close proximity to cherry fruits, even if contests can also occur on host plant leaves [46,47,48,52,60].

In some tephritid species, prior residence at a given site substantially enhances the male’s chances to win a fight. Good examples are *A. ludens* [18,61], *B. oleae* [21] and *R. completa* [86]. Furthermore, Ramos [87] noted that in wild Costa Rican *C. capitata*, resident males won over 80% of the aggressive encounters. In contrast, in Hawaiian *C*. *capitata*, male intruders won more than two-thirds of all aggressive interactions with resident males [20]. In a further study on Hawaiian medfly males [88], prior residency did not appear to provide any advantage; the same was also shown in *B. dorsalis* [67].

### 2.4. When Males Fight

Detailed information about diel dynamics of male-male aggressions in Tephritidae is not abundant. Most of the observations on aggressive behavior have been carried out alongside courtship and mating studies. Few details have been provided about the time of observation of aggressions within each day:(*i*) no information: *Procecidochares* sp. [64], *P. gentilis* [68], 48 tephritid species [53], *R. indifferens* and *R. pomonella* [48], *C. capitata*, [88]; (*ii*) early morning-late afternoon: *R. indifferens* [47],* T. bisetosa* [70],* T. curvicauda* [55], *A. ludens* [18], *A. trixa*,* V. dodsoni* [63], *C. capitata* [65],* B. oleae* [21]; (*iii*) late afternoon: *A. suspensa* [61]; *(iv)* 2–3 h before dusk: *C. capitata* [67]; and (*v*) immediately before dusk: *B. cucurbitae* [17].

Most importantly, no comparisons have been carried out about the magnitude of male-male contests over different daylight periods, with two exceptions, *A. ludens* and *B. oleae* [18,21]. In indoor habitat cages, *A. ludens* fighting males display a comparable number of wing waving acts in the daily pre-sex period than the sex period [18]. Concerning the olive fruit fly, the number and mean duration of male-male contests has been found higher in the late morning than the afternoon [21], although this species mainly mates at dusk [31,89].

Lastly, in a number of tephritid species, site fidelity after aggressive interactions, seems to be restricted to a short time period, ranging from some minutes to a few hours. Good examples are *B. oleae*, [21], *A. trixa*, *V. dodsoni* [63], *R. indifferens* [47,48], while other species of composite-infesting tephritids have been described as territorial on their host plants for longer periods [53,90,91,92].

## 3. Female-Female Fighting Behavior

### 3.1. Why Females Fight

**Figure 1:**
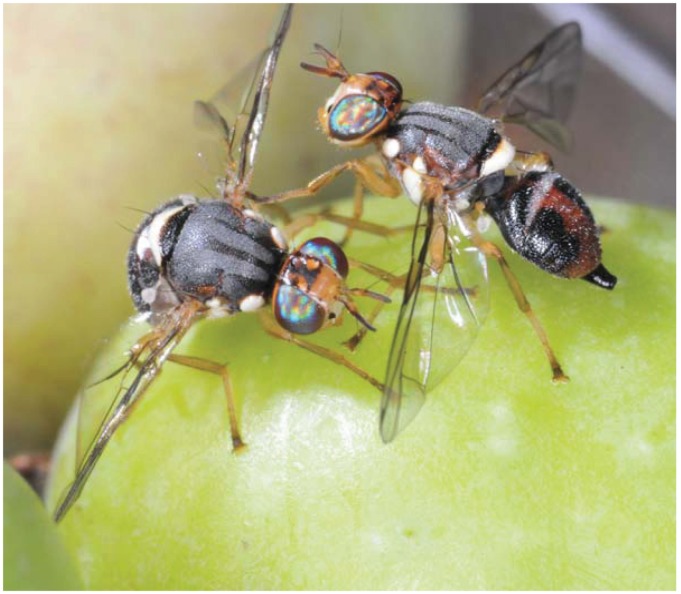
Females of the olive fruit fly, *Bactrocera oleae*, boxing for an olive fruit (adapted with permission from [21]).

Current theory says that tephritid females have two main behavioral tools to maintain single oviposition sites, thus increasing the chances of their eggs developing successfully. First, the host marking behavior, reported for at least 27 species (see Benelli* et al.* [11,12] for dedicated reviews), Second, female-female contests for oviposition sites. The latter are widespread among tephritids. Good examples are *A. ludens* [18], *B*. *dorsalis* [93], *B. invadens* [62], *B. oleae* (Figure 1) [21], *B. tryoni* [94], *C. capitata* [65,95], *P. gentilis* [68], *R. indifferens* [47], and *T. bisetosa* [70].

Particularly, for small fruits, maintenance of single oviposition sites is of crucial importance, since more than one oviposition event per fruit may lead to larval competition for resources, thus reducing larval survival and growth rate [47,96,97].

### 3.2. How Females Fight

Female-female combats are usually composed of aggressive displays similar to those observed for male-male fights. Basically, they rely on wing waving, chasing, pouncing and boxing acts [11,53]. Well-known examples are *A. ludens* [18], *B*. *dorsalis* [93], *B. invadens* [62], *B. oleae* (Figure 1) [21], *B. tryoni* [94], *P. gentilis* [68], *R. indifferens* [47], *R. pomonella* [46], and *T. bisetosa* [70]. *C. capitata* females use wing strikes, head pushing and dives, following the same sequence of fighting males [65,66]. Also in *T. curvicauda* wing strike has been observed between fighting females [55].

### 3.3. Where Females Fight

In frugivorous Tephritidae species, female-female contests usually occur on host plant fruits (e.g., *A. ludens* fights on *Citrus* spp. [18], *B. oleae* on olives [21], *C. capitata* on coffee berries [97], *R. indifferens* on cherries [47] and *R. pomonella* on apples [46]), although they have been reported also on host plant leaves and walls of laboratory cages [18,21,65]. A similar trend has been observed for non-frugivourous species. For instance, in *T. bisetosa* the ovipositing female does not allow other females on “her” sunflower head, and fights them off with wing waving and boxing [70].

### 3.4. When Females Fight

As discussed for male-male fights, information about daily dynamics of aggression in tephritid females is scarce. To the best of my knowledge, only two studies have quantified the magnitude of female-female contests over daylight periods. In *B. oleae*, the number of female-female aggressions is higher in the late morning than in the afternoon, with no differences in fighting duration [21]. Similarly, in* A. ludens*, female-female aggressive interactions occurred mostly on fruits before the sexual activity period [18].

## 4. Interspecific Contests in Tephritidae

Aggressions can also occur between different Tephritidae species sharing the same habitat. These interactions may play a key role in competitive displacement of one species against another (see [98] for a review). For instance, interspecific male-male contests (*i.e.*, wing waving, chasing, pouncing, and boxing) for fruits have been observed between the western cherry fruit fly, *R. indifferens*, and the apple maggot, *R. pomonella* [48]. A more recent example concerns the invasive fly *B. invadens* displacing *C. cosyra* in mango orchards [62]. Here, competitive displacement is routed by two mechanisms, adult aggressive interactions (both sexes) and larval scrambling for resources [62]. In this scenario, adult body size could be considered as a potential predictor of the success of the invader fly [98], since this trait affects fighting outcomes in several tephritid species [38,64]. In addition, in several tephritid species both sexes exhibit wing waving, pouncing and boxing acts also during aggressive or defensive behavior towards other arthropods such as predators (e.g., *A. ludens* against Salticidae spiders) [99,100]. Overall, knowledge about interspecific aggressive interactions in Tephritidae is still scarce, with special reference to cues evoking aggression, and further work is needed, since this information is helpful in predicting pest population dynamics on a given crop.

## 5. Knowledge on Aggression in Tephritidae: Future Directions and Potential Implications for Integrated Pest Management

**Figure 2:**
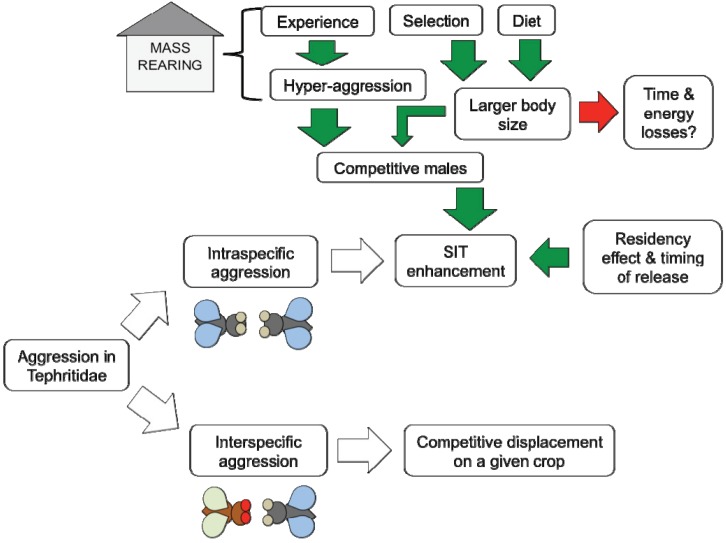
How knowledge on aggressive behavior in Tephritidae flies may be exploited to enhance behavior-based control tools. Dynamics beneficial to pest control are the green arrows, while detrimental mechanisms are the red ones.

As regards Integrated Pest Management of tephritid pests, the Sterile Insect Technique (SIT) is one of the most reliable non-disruptive control tools. However, it has two major limitations. First, it is only effective when integrated on an area-wide basis [101]. Second, sterile males have lower mating competitiveness over wild ones, due to mass-rearing procedures as well as to damage/stress occurring during sterilization, shipping, and release [102]. Information about male-male conflicts in Tephritidae may be important in enhancing mating competitiveness of SIT males against wild ones (see also [11,12]). Knowledge on aggression can contribute to mass-rearing optimization in SIT programs, mainly via manipulation of social experience, selection, and diet (Figure 2).

### 5.1. Experience

In a number of arthropod species experiencing aggression affects performance in subsequent contests. Current theory predicts that, after an intra-specific aggressive interaction, winners are more likely to win again and losers more likely to lose again [29,103,104,105]. However, studying the olive fruit fly, it has been observed that both winners and losers of two consecutive encounters displayed higher intensity of aggression and fought longer in subsequent contests. The enhanced fighting performance of winners and losers is affected by merely experiencing a contest, while the relative outcome or involvement in a physical fighting experience does not affect fighting skills [106]. This raises the possibility of inducing hyper-aggression in SIT males by manipulating the density of the flies in cages, thus increasing the number of male-male contests, and providing small potted host plants to create a semi-field environment during the pre-release phase. This may help sterile males to refine their fighting skills and achieve better subsequent contest outcomes against wild ones (Figure 2).

### 5.2. Selection

Boake* et al.* [107] stated that radiation doses should be calibrated to ensure that SIT males are not behaviorally weakened. However, Boake* et al.* [107] also pointed out that mass-rearing should select against male aggressive tendencies, due to anecdotal evidence from *Drosophila silvestris* Basden (Diptera: Drosophilidae) suggesting that highly aggressive males may be aggressive toward females as well as toward other males, thus reducing their mating success. This point deserves further experimental work. To my mind, selection against male intra-sexual aggression is not appropriate, since the majority of tephritid species (including the most important pest genera *Anastrepha*,* Bactrocera* and *Ceratitis*), have a non-resource-based lek polygyny, in which both male intra-sexual selection and inter-sexual selection are crucial to ensure reproductive success [101]. Furthermore, the observations reported above may be due to misleading identification of courtship signals as aggressive displays [e.g., wing vibration in *C. capitata* is a courtship display (*sensu* Briceño* et al.* [108], but was reported as a component of aggressive behavior [65,66]). However, I cannot exclude that positively-selected/induced hyper-aggression in males may lead to detrimental effects, due to time and energy losses in contests, thus reduced mating success (Figure 2).

### 5.3. Diet

In several Tephritidae species, body size is a key factor affecting fighting success in males (e.g., [64]) and females (e.g., [93]). Manipulation of larval diet composition, with special reference to protein content, can help to obtain robust sterile males, with larger body size and enhanced chances to win territorial contests in the field (Figure 2).

### 5.4. Residency Effect

A last point worth mentioning is the relationship between residency and fighting success. It has been shown for a number of tephritid pests that prior residents are more likely to win contests [18,21,61,86]. Thus, the precise timing of the mass-release of SIT males might allow them to establish territories and gain fighting experience before the time of day in which the species usually mates (e.g., dusk for the olive fruit fly). This may be particularly useful for monogamous/oligogamous species.

## 6. Conclusions

This review highlights a noteworthy variation in aggressive behavior among Tephritidae species, implying that physiological, ecological, and evolutionary issues of species-specific aggressive behavior should be investigated in greater depth. Four neglected issues particularly deserve further research efforts: the diel periodicity of male-male aggressive behavior, the cues evoking aggression, the role of previous experience on fighting performances and the evolution of behavioral lateralization of aggression. Overall, we really need to know more about how, where, when and why aggression occurs in true fruit flies, since a further array of practical implications may arise from this knowledge. Information of intraspecific aggressive behavior may help to improve competitiveness of SIT males, manipulating social experience, selection, and diet. This is of particular importance for species with male dominance polygyny. Knowledge on interspecific aggressive interactions leading to competitive displacement in Tephritidae could be helpful to predict pest population dynamics on a given crop.
